# A review of pregnancy counseling with abnormal fetuses

**DOI:** 10.1192/j.eurpsy.2021.2210

**Published:** 2021-08-13

**Authors:** S. Haqshenas

**Affiliations:** Reproductive Health And Midwifery, Mazandaran University of Medical Sciences, Sari, Iran

**Keywords:** congenital anomalies, psychological counseling, prenatal counseling

## Abstract

**Introduction:**

This review study examines the cases of improving the therapeutic skills of therapists and areas of counseling and the important cases that midwives have to provide services and manage conditions if Diagnosis of an abnormal fetus requires attention.

**Objectives:**

We aim to find the best ways of counseling for helping parents with diagnosed abnormal fetuses

**Methods:**

A search conducted by using the keywords congenital anomalies, psychological counseling, prenatal counseling in PubMed, science direct, clinical key and Google scholar search engine. after screening, the complete data of 20 articles were included in this review article.

**Results:**

The results showed that pregnancy counseling with abnormal fetuses includes medical and psychological counseling. In medical counseling, knowledge of the types of tests and their interpretation is important, and prenatal screening training programs for health care providers should be revised based on their educational needs. In psychological counseling, to meet the needs of a changing population of clients Midwives in the context of the wider healthcare system need accurate knowledge of religious beliefs and cultural contexts of their clients in order to take the best approach to relevant care. The occurrence of a diagnosis of congenital anomaly during transmission to parents adds to the accumulation of stress-related events that may increase the risk of developing psychological symptoms in the early stages after diagnosis.

**Conclusions:**

Considering the different cultures of different countries of the world, midwifery counseling skills play an important part in the diagnostic and therapeutic process. Therefore, creating extraordinary educational programs on university education is needed for midwives.

**Disclosure:**

No significant relationships.

